# Mycoplasma pneumoniae and Bronchiolitis Obliterans: How a Common Organism Leads to a Rare Pulmonary Disease in Pediatrics

**DOI:** 10.7759/cureus.17193

**Published:** 2021-08-15

**Authors:** Antonella Jimenez, Wilfredo De Jesús-Rojas

**Affiliations:** 1 Department of Pediatrics, San Juan Bautista School of Medicine, Caguas, PRI; 2 Department of Pediatrics, University of Puerto Rico School of Medicine, San Juan, PRI; 3 Department of Pediatrics, Ponce Health Sciences University, Ponce, PRI

**Keywords:** bronchiolitis obliterans, mycoplasma pneumoniae, rare lung disease, lung cavitation, lung abscess, pediatrics

## Abstract

A rare lung disease, bronchiolitis obliterans (BO) is characterized by airway obstruction and fibrosis of the terminal and respiratory bronchioles. It usually occurs after lung and bone marrow transplants, hematopoietic stem cell transplantation (HSCT), inhalation of toxins, respiratory infections, or in association with several different connective tissue or irritable bowel diseases. When BO is caused by an infection it is referred to as post-infectious bronchiolitis obliterans (PIBO). The prevalence of BO is unknown but has been seen to occur worldwide. The pathophysiology of BO is not fully understood but there is evidence of fibroproliferation leading to abnormal airway remodeling with inflammatory mediators and granulation tissue that narrows the bronchial lumen. Diagnosis of BO is achieved via a combination of clinical manifestations, patient history, pulmonary function test (PFT), radiological imaging, and lung biopsy as the gold standard. Since there is limited literature on pediatric cases with BO and diagnosis may sometimes be challenging, we aim to bring awareness to a pediatric case where PIBO developed after a common pulmonary infection of *Mycoplasma pneumoniae*.

## Introduction

*Mycoplasma pneumoniae* is a bacterium that causes upper respiratory infection and is the most common cause of atypical pneumonia in the United States [1]. In many patients, infection is usually self-limited, asymptomatic, or mild. Symptomatic patients may develop a low-grade fever, headache, malaise, wheezing, and/or a dry persistent cough that can be treated with antibiotics [1]. Since the organism lacks a cell wall, does not gram stain, and requires special prolonged culturing techniques, it is not routinely cultured in outpatient settings [1]. It is unusual for patients to develop severe respiratory disease requiring mechanical ventilation and only 18% of cases require hospitalization [2]. Despite this, rare complications such as pleural effusion, acute respiratory distress syndrome (ARDS), pulmonary abscess, bronchiolitis obliterans (BO), respiratory failure, or extrapulmonary complications can develop [1]. Children, the elderly, and immunocompromised patients are most susceptible to complications that can result in irreversible lung damage. 

We present a case of a generally healthy 15-year-old male who developed lung cavitation and BO after a past infection with *Mycoplasma pneumoniae* in Puerto Rico. The case is unusual because severe irreversible airway obstruction developed after the bacterial infection that may have occurred months to years before the diagnosis of BO. The case demonstrates the importance of early identification, diagnosis, and treatment of *Mycoplasma pneumoniae* and BO in children who may be at greater risk of complications and irreversible lung damage. 

## Case presentation

A 15-year-old male with a history of sinusitis and urticaria presented to the emergency department with dyspnea and chest pain for one day prior to evaluation. Past medical history revealed that the patient was born full-term, had no drug allergies, denied exposure to smoke, and was up-to-date with immunizations. The patient reported no history of recurrent hospitalization but his mother recalled that during childhood the patient had a past infection with *Mycoplasma pneumoniae*; further data was not available. The only pertinent positive on physical examination was the presence of bilateral polyphonic wheezes across his chest with a prolonged expiratory phase. Laboratory studies were negative for *Histoplasma*, tuberculosis, *Aspergillus fumigatus*, rheumatoid factor, antinuclear antibodies (ANA), and HIV. He was also negative for mutations in the cystic fibrosis transmembrane conductance regulator (CFTR) gene and had a negative sweat test. Total IgA, IgM, IgG, as well as IgG subclasses, were all within normal limits. *Mycoplasma pneumoniae*-IgG titer was elevated at 3.43 (reference range (>1.10: positive) and IgM titer was normal at 382 U/mL (negative: <770 U/mL). A bronchoscopy was performed showing normal airway anatomy, normal segmental distribution, and no tracheobronchomalacia observed. Bronchoalveolar lavage (BAL) was negative for signs of infections including bacteria, fungi, respiratory syncytial virus (RSV), adenovirus, acid-fast bacilli (AFB), *Mycoplasma*-IgM, and galactomannan. No malignant cells or hemosiderin-laden macrophages were observed. Cell counts in BAL showed 55% macrophages, 38% lymphocytes, and 7% neutrophils. 

**Figure 1:**
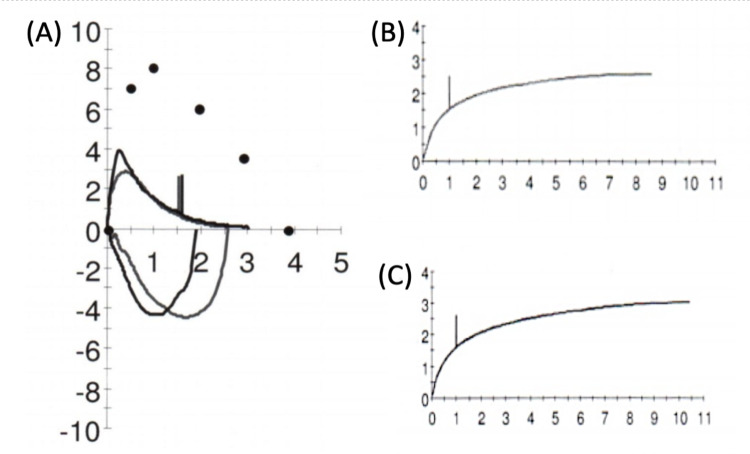
(A) Post hospital discharge baseline PFT flow-volume loop showing a characteristic 'scooping' of the expiratory curve, which may be suggestive of lower airway obstruction as seen in BO; (B) Pre-bronchodilator volume-time curve and (C) Post-bronchodilator volume-time curve are presented with no significant changes PFT: Pulmonary function test

Shortly after discharge, the patient had a complete pulmonary function test (PFT) that was indicative of a severe obstructive airflow pattern despite being asymptomatic. Forced expiratory volume in one second (FEV_1_) was 43% of predicted, forced vital capacity (FVC) was 67% of predicted, and FEV_1_/FVC ratio was 64% of predicted. No significant improvement in FEV_1_ was noted after the bronchodilator challenge (Table 1). His flow-volume loop and time-volume graph were also consistent with severe pulmonary obstructive lung disease (Figure 1). 

**Table 1:** PFT results approximately one week and one year post hospital discharge. No significant changes on PFT were noted at the one-year mark. Severe obstructive airflow pattern with negative bronchodilator challenge test was evident. TLC: 82%, RV: 118%, RV/TLC: 129%, DLCO: 120%, DLCO/VA or DL/VA: 181% FEV_1_: Forced expiratory volume in one second; FVC: Forced vital capacity; FEF: Forced mid-expiratory flow; PEF: Peak expiratory flow; PFT: Pulmonary function test; RV: Residual volume; TLC: Total lung capacity; DLCO: diffusing capacity of the lungs for carbon monoxide; VA: alveolar volume

Pulmonary function test (PFT)	Approximately one week post-discharge			Approximately one year post-discharge
	Baseline/pre-bronchodilator (% Predicted)	Post-bronchodilator (% Predicted)	Percentage of change (% predicted)	Baseline/pre-bronchodilator (% predicted)
FEV1	43	47	+9	42
FVC	67	80	+19	65
FEV1/FVC	64	59	-8	64
FEF 25-75%	15	18	+14	15
PEF max	43	47	+10	47

Initial chest X-ray studies showed a right upper lobe (RUL) cavitation. The patient was diagnosed with a pulmonary abscess and treated with ceftriaxone and clindamycin. Approximately one month later, an enhanced CT of the chest showed bilateral mosaic attenuation pattern with an interval decrease in the size of the abscess. The patient was discharged and follow-up with a pediatric pulmonologist was completed. After analysis of the patient’s clinical presentation, PFT results, laboratory and radiological findings, a diagnosis of post-infectious bronchiolitis obliterans (PIBO) was made because of prior history of *Mycoplasma* infection with elevated IgG titers during hospital admission (Table 2). 

**Table 2:** Main criteria used for the diagnosis of BO compared to the findings in this patient. Lung biopsy was not performed due to the invasive nature of the procedure PFT: Pulmonary function test; HRCT: High-resolution CT, DLCO: Diffusing capacity of the lungs for carbon monoxide; FEV_1_: Forced expiratory volume in one second; FVC: Forced vital capacity, TLC: Total lung capacity; BO: Bronchiolitis obliterans

Criteria for diagnosis of bronchiolitis obliterans (BO)			
	Standard criteria for diagnosis of BO		Patient findings
PFT		Airflow obstruction that is irreversible with bronchodilator challenge, reduced FEV_1 _<80%, reduced FEV_1_/FVC ratio <0.80, DLCO is usually reduced but can be normal, increased TLC with air trapping/hyperinflation	Severe airflow obstruction irreversible with bronchodilator challenge, reduced FEV_1_ <80%, reduced FEV_1_/FVC ratio <0.80, DLCO was normal at 120%
Chest CT/HRCT		Bronchial wall thickening, mosaic attenuation pattern/tree-in-bud formation, air trapping, atelectasis, or bronchiectasis	Bilateral mosaic attenuation pattern
Physical exam & symptoms		Decreased breath sounds, prolonged expiratory phase, dyspnea, cough, wheezing, or rales	Bilateral polyphonic wheezes, prolonged expiratory phase
Surgical lung biopsy		Thickening and fibrosis of the peribronchiolar submucosa, narrowing/obstruction of the bronchial lumen, macrophage and lymphocytic infiltrates, myofibroblast and fibroblast accumulation, collagen, and granulation tissue deposition	Not performed

**Figure 2:**
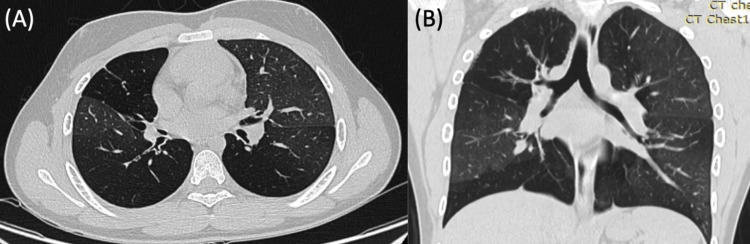
(A) Axial HRCT of the chest shows bilateral mosaic attenuation pattern approximately ten months after symptom onset; (B) Coronal HRCT with stable pulmonary disease post-FAM treatment approximately one year later showing similar mosaic attenuation pattern, without improvement HRCT: High-resolution CT; FAM: Fluticasone oral inhalation, azithromycin, montelukast

The patient was subsequently started on fluticasone oral inhalation, azithromycin, and montelukast (FAM) treatment, which he continued for slightly over one year. PFT was repeated after one year of FAM treatment, but no improvement was noted as compared with baseline (Table 1). Comparison of an axial high-resolution CT (HRCT) of the chest before FAM treatment (Figure 2, A) and coronal HRCT of the chest after FAM treatment (Figure 2, B) continued to show areas of a mosaic pattern consistent with BO with no significant changes. At this point, the FAM trial was discontinued due to a lack of improvement in the patient’s clinical status, imaging studies, and pulmonary function. 

Due to the association between BO and gastroesophageal reflux as a known risk factor particularly in bronchiolitis obliterans syndrome (BOS), gastrointestinal imaging studies were performed. A small sliding-type hiatal hernia was identified without any evidence of gastroesophageal reflux. Additionally, the patient underwent primary immunodeficiency genetic testing and several variants were identified in the ataxia telangiectasia mutated (ATM), dedicator of cytokinesis 8 (DOCK8), lipopolysaccharide-responsive and beige-like anchor protein (LRBA), mucosa-associated lymphoid tissue lymphoma translocation protein 1 (MALT1), and Ras-related C3 botulinum toxin substrate 2 (RAC2) genes (Table 3). However, all variants were classified as having uncertain significance by ClinVar databases and none have been associated with BO in the literature. 

**Table 3:** Primary immunodeficiency panel shows genetic variants in our patient. None of these variants have been reported in individuals known to have a gene-related disorder. Available evidence and algorithm studies are currently insufficient to determine the role of the variant in disease, therefore they have been classified as VUS VUS: Variant of uncertain significance; ATM: Ataxia telangiectasia mutated; DOCK8: Dedicator of cytokinesis 8; LRBA: lipopolysaccharide-responsive and beige-like anchor protein; MALT1: Mucosa-associated lymphoid tissue lymphoma translocation protein 1; RAC2: Ras-related C3 botulinum toxin substrate 2

Gene	Variant	Zygosity	Classification
ATM	c.4724G>A (p.Arg1575His)	heterozygous	VUS
DOCK8	c.431_432delinsTT (p.Gly144Val)	heterozygous	VUS
LRBA	Gain (Exons 2-35)	Copy number = 3	VUS
MALT1	c.202C>A (p.Arg68Ser)	heterozygous	VUS
RAC2	c.88G>A (p.Gly30Arg)	heterozygous	VUS

## Discussion

PIBO is a rare pulmonary disorder that may be a sequela of common viral and bacterial infectious processes like *Mycoplasma pneumoniae*. A high index of suspicion is needed to differentiate BO from other etiologies. For example, classic signs such as persistent dyspnea, cough, wheezes, airflow limitation unresponsive to bronchodilator challenges, and mosaic pattern on HRCT of the chest are important clinical clues for BO diagnosis. Although lung biopsy is the gold standard for the diagnosis of BO, it is an interventional procedure necessary when non-classic manifestations of BO are observed. Taking into consideration the clinical manifestations of our patient along with his elevated levels of *Mycoplasma* IgG, a diagnosis of PIBO was made likely due to prior history of *Mycoplasma pneumoniae* infection of an unknown timeframe. Since PIBO can develop months to years after the initial respiratory illness, it is difficult to pinpoint when the initial infection occurred in our patient [3]. This can explain why the patient's *Mycoplasma pneumoniae*-IgM antibody levels were normal at the time of admission. It is also likely that the presence of the abscess in this patient was a complication of PIBO. In this case, the patient had a mosaic pattern on radiological images and spirometry showing obstructive airflow patterns without improvement after the bronchodilator test. Our patient's results matched with his dyspnea, chest pain, bilateral wheezing upon hospitalization, and past Mycoplasma exposure fit the diagnosis of PIBO secondary to a past Mycoplasma infection. 

*Mycoplasma pneumoniae* is a bacterium that commonly causes community-acquired pneumonia. It attaches via adherence proteins to the respiratory epithelium, damaging the cilia, and causing epithelial sloughing [1]. An important virulence factor of *Mycoplasma* is the community-acquired respiratory distress syndrome (CARDS) toxin, which has been shown to increase cytokine production and cause airway hyperreactivity closely resembling asthma [4]. In animal models with *Mycoplasma*, CARDS toxin levels correlated with disease severity [4]. Infection with *Mycoplasma* can be asymptomatic or typically mild and slowly resolves without requiring hospitalization, but if severe enough or left untreated it can lead to complications. In approximately 10% of all cases, relapse occurs that can be confirmed radiologically by lung infiltrates [5]. Lung abnormalities such as pneumatocele development, bronchiectasis, abscess formation, lung cavitation, and BO though rare, have been reported after *Mycoplasma* infection [5]. PIBO has classically been associated with adenovirus serotypes 3, 7, and 21 and RSV in children, but some studies have also shown PIBO due to *Mycoplasma* infection. In a study of 42 pediatric cases of PIBO, the etiology was mostly due to adenovirus (50%) followed by *Mycoplasma Pneumoniae* (24%) with a lesser proportion of cases due to RSV, *Chlamydia pneumoniae*, influenza A or B, and *Legionella pneumophila* [6]. Therefore, physicians must be aware of the potential complication of BO that can result after *Mycoplasma* infection, especially in children in geographic locations with higher incidence rates. 

A unique aspect of this case was the presence of lung cavitation, which was the reason for our patient to seek medical attention. Lung cavitation is commonly caused by infection with gram-positive or negative bacteria. The organism may enter the airways evading host defenses causing necrotizing pneumonia or lung abscess [7]. Lung abscess is more commonly reported after *Bacteroides fragilis*, *Mycobacterium tuberculosis*, *Streptococcus pneumoniae*, *Haemophilus influenzae*, *Klebsiella pneumoniae*, *Staphylococcus aureus*, which is the most commonly isolated pathogen of lung abscess in children [8]. Almost all cases of lung abscess are due to polymicrobial flora infection [8]. Other causes of lung cavitation include non-infectious processes such as lung carcinoma, lymphoma, sarcoidosis, Wegener’s granulomatosis, or Langerhans cell histiocytosis [2]. Lung cavities due to *Mycoplasma pneumoniae* are extremely rare but have been reported particularly in immunocompetent patients. Kashif et al. showed a patient with a cavitary lesion caused by *Mycoplasma pneumoniae* that eventually resolved after a three-month follow-up period with levofloxacin [2]. Yet unlike in our case, the patient did not show any symptoms or signs of BO. As such, although *Mycoplasma pneumonia* is not commonly associated with lung cavitation, rare cases may still occur. 

Another important factor to consider is the association between lung cavitary lesions and bronchiolitis obliterans organizing pneumonia (BOOP) also known as cryptogenic organizing pneumonia (COP). BOOP and BO are similar disease processes, but BOOP pathogenesis is mainly localized to the alveoli while BO pathogenesis is localized to the terminal bronchioles. BOOP often presents as solitary pneumonia with single or multiple masses, nodules, or cavitary lesions while BO is less likely to be associated with pulmonary cavitation [9]. BOOP is an inflammatory response to mild alveolar damage. Gaps form in the basal lamina that allow interstitial fibroblast cells to migrate into the alveolar lumen where they deposit granulation tissue. In contrast, BO consists of inflammation and fibroproliferation within the bronchioles. BOOP can result from chronic sequelae of previous inflammation or infection that is no longer present and is therefore referred to as idiopathic [9]. Since there is evidence of *Mycoplasma* infection causing BOOP with cavitary lesions, this differential diagnosis was considered for our patient [10]. Additionally, BOOP and BO differ in treatments and outcomes, so a clear understanding of each disease is necessary to provide the best clinical management for patients. 

Radiological imaging for BOOP and BO can often be nonspecific but generally, BOOP presents as patchy alveolar consolidations with ground-glass opacities, infiltrates, cavitation, and/or nodules. However, in BO, ground-glass opacities are very scarce, and radiologic imaging shows a mosaic attenuation pattern, hyperinflation, air trapping, tree-in-bud formations, and/or bronchiectasis in advanced cases [3]. PFT also differs as patients with BO have an obstructive airflow pattern on spirometry while patients with BOOP typically have a restrictive airflow pattern. Additionally, corticosteroid treatment used to treat BOOP has been shown to eliminate inflammatory nodules without any evidence of recurrence [9]. On the other hand, corticosteroid treatment for BO is often more complex and controversial due to its limited benefits and adverse systemic effects [11]. Therefore, distinguishing between BOOP and BO is necessary to identify the best treatment option. In our patient case, even though he had a cavitary lesion, which is typically more commonly seen with BOOP, his obstructive airflow pattern on PFT and mosaic attenuation pattern on radiologic imaging indicated BO or more specifically PIBO, as a more definitive diagnosis. 

Treatment options for PIBO are very limited and none have been shown to cause definitive improvement. In some cases, oral, inhaled, or intravenous corticosteroids such as prednisone, budesonide, or pulse methylprednisolone therapy have been used to reduce the inflammatory damage in the airways [11-14]. Other immunosuppressive drugs such as tacrolimus or cyclosporine have been used for patients with BOS [15]. Antibiotics such as azithromycin have been shown to improve lung function and reduce the incidence of BOS [3,14]. When fluticasone oral inhalation, azithromycin, and oral montelukast are combined (referred to as FAM), it has been shown to prevent the decline of lung function in patients with BOS [14,15]. Anti-inflammatories such as infliximab, chloroquine, and hydroxychloroquine have shown minimal improvement in small studies [13]. Finally, bronchodilators such as terbutaline and ipratropium bromide have been shown to improve symptoms in some cases of BO [14]. There is some evidence that systemic corticosteroids and oral azithromycin can be effective in some cases of PIBO, but these studies are few and limited [6]. Additionally, past studies have reported that immediate treatment is important in order to observe beneficial effects before fibrosis and scarring have occurred. Despite these data, further research is necessary to determine more specific and effective treatments for PIBO. It is also necessary to elucidate why some therapies are more effective in some cases of BO (specifically after lung or hematopoietic stem cell transplantation) or BOOP, which may be related to differences in pathogenesis, the inflammatory mediators involved, and variations between patients' immune responses. 

## Conclusions

BO is a rare lung disease that can have serious irreversible consequences for patients especially since effective treatment options are scarce and may not be as effective in later stages of the disease. This case report aims to bring awareness to PIBO especially after an untreated *Mycoplasma pneumoniae* infection in a pediatric patient. Physicians must therefore be vigilant of the potential complication of BO and lung cavitation that can result after common infectious etiologies, especially in children in geographic locations with higher incidence rates of *Mycoplasma*. Early identification and treatment of PIBO may improve patient outcome, symptoms, and avoid further complications. 
